# Functional ligands for improving anticancer drug therapy: current status and applications to drug delivery systems

**DOI:** 10.1080/10717544.2022.2089296

**Published:** 2022-06-28

**Authors:** Rajiv Bajracharya, Jae Geun Song, Basavaraj Rudragouda Patil, Sang Hoon Lee, Hye-Mi Noh, Da-Hyun Kim, Gyu-Lin Kim, Soo-Hwa Seo, Ji-Won Park, Seong Hoon Jeong, Chang Hoon Lee, Hyo-Kyung Han

**Affiliations:** College of Pharmacy, Dongguk University-Seoul, Goyang, Korea

**Keywords:** Drug delivery, target selectivity, cell surface receptors, cell penetrating peptides, tight junction opening, anticancer

## Abstract

Conventional chemotherapy lacking target selectivity often leads to severe side effects, limiting the effectiveness of chemotherapy. Therefore, drug delivery systems ensuring both selective drug release and efficient intracellular uptake at the target sites are highly demanded in chemotherapy to improve the quality of life of patients with low toxicity. One of the effective approaches for tumor-selective drug delivery is the adoption of functional ligands that can interact with specific receptors overexpressed in malignant cancer cells. Various functional ligands including folic acid, hyaluronic acid, transferrin, peptides, and antibodies, have been extensively explored to develop tumor-selective drug delivery systems. Furthermore, cell-penetrating peptides or ligands for tight junction opening are also actively pursued to improve the intracellular trafficking of anticancer drugs. Sometimes, multiple ligands with different roles are used in combination to enhance the cellular uptake as well as target selectivity of anticancer drugs. In this review, the current status of various functional ligands applicable to improve the effectiveness of cancer chemotherapy is overviewed with a focus on their roles, characteristics, and preclinical/clinical applications.

## 1. Introduction

Surgery, radiotherapy, and chemotherapy are the standard methods in clinical practice for the treatment of cancers. Among them, chemotherapy can be used alone as the first-line treatment, or it can be used in combination with other approaches (Atlihan-Gundogdu et al., 2020; Chen et al., 2020). For example, chemotherapy is used to reduce the size of tumors prior to surgery, and it can be employed to prevent the proliferation of cancer cells after surgery or radiotherapy (Amjad et al., 2021; Bajpai et al., 2021). Although these conventional methods achieved clinical success, to a certain extent, in improving the survival rates of cancer patients, they exhibited critical issues with nonspecific tissue distribution of anticancer drugs to both healthy and pathological cells (Yetisgin et al., 2020). Given that nonspecific drug delivery leads to multiple issues, including severe side effects, insufficient drug distribution to the targeted organs, and rapid elimination from the bloodstream (Manzari et al., 2021), it ultimately limits the effectiveness of chemotherapy. This is a major drawback of conventional chemotherapy and thus it is highly demanded to deliver anticancer drugs selectively to the intended site of action (Baig et al., 2021; Gupta & Kim, 2021; Mitchell et al., 2021).

Various approaches have been extensively explored for target-selective delivery of anticancer drugs (Cho, 2020; Zhao et al., 2020; Gupta & Kim, 2021). One of them is the utilization of functional ligands that can interact with specific receptors overexpressed in malignant cancer cells. Various functional ligands including folic acid, hyaluronic acid, transferrin, peptides, and antibodies, have been extensively examined to develop tumor-selective drug delivery systems (Zhao et al., 2020). Furthermore, cell-penetrating peptides or ligands for tight junction opening in tumors are also actively pursued to improve the intracellular delivery of anticancer drugs. These functional ligands are used alone or in combination to improve intracellular uptake as well as target selectivity of anticancer drugs. In this review, recent approaches for improving the effectiveness of chemotherapy are covered with a focus on the application of various functional ligands. In the following sections, target-selective drug delivery systems are discussed, with particular emphasis on the application of function ligands that can interact with specific receptors overexpressed in cancer cells. In addition, functional ligands applicable to enhancing intracellular uptake of anticancer drugs are covered in light of their roles, characteristics, and applications in designing anticancer drug delivery systems.

## 2. Approaches for target-specific drug delivery

### 2.1. Passive targeting

**Figure 1.**
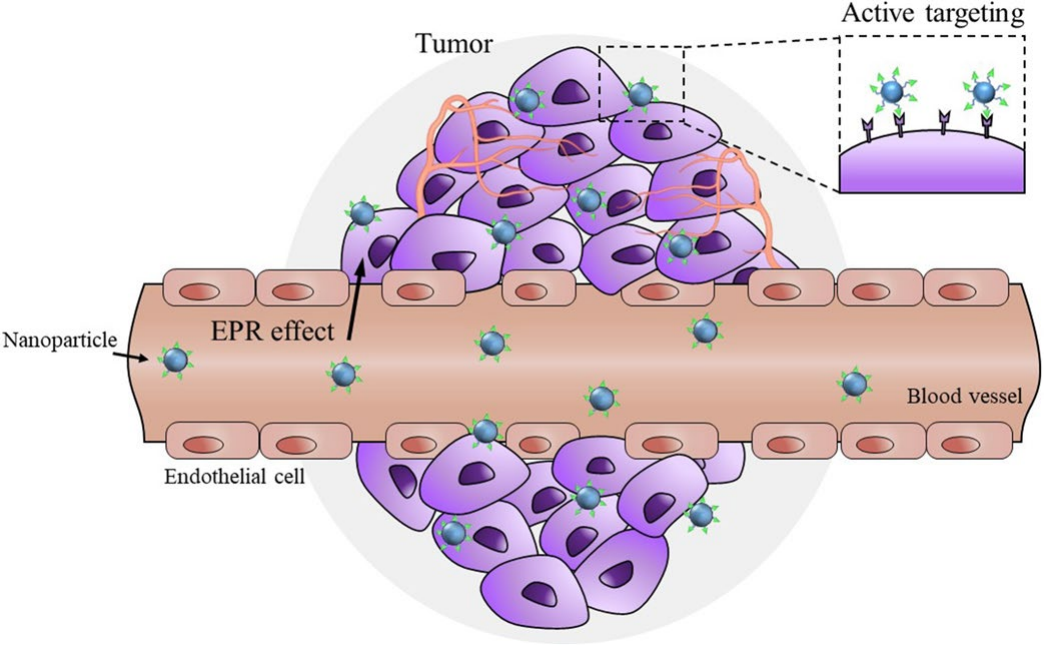
Tumor-targeted drug delivery via EPR effect and ligand-recognition. Nanoparticles can be accumulated more in tumors than in normal tissues due to leaky tumor vasculatures and poor lymphatic drainage of tumors (EPR effect). While passive targeting is based on EPR effect, active tumor targeting is based on the selective interaction of ligand-coated nanoparticles with specific receptors overexpressed in tumor cells. The extent and intensity of EPR effect in human tumors is highly debated and active targeting is preferred for tumor-selective drug delivery.

Passive targeting is the selective accumulation of nano-drug delivery systems (nano-DDS) at the desired site due to pharmacological or physicochemical factors (Attia et al., 2019). Passive targeting approaches are extensively employed in delivering drugs to angiogenic tissues such as tumors. Nano-DDS for passive targeting make use of the anatomical and functional differences between the normal and tumor vasculature to penetrate into the disorganized and fenestrated tumor site (Manzari et al., 2021). In comparison to free drugs, nano-DDS have hydrodynamic diameters exceeding the renal clearance threshold and have prolonged circulation in the blood, which leads to extravasation from leaky tumor vessels (Golombek et al., 2018; Subhan et al., 2021). The poor lymphatic drainage in solid tumors leads to the enhanced accumulation and retention of nano-DDS at the tumor site (Figure 1). This is popularly referred to as the enhanced permeability and retention (EPR) effect and is exploited in the treatment of diverse tumors (Golombek et al., 2018). Various organic and inorganic nano-DDS including liposomes, polymer − drug conjugates, polymer micelles, PEGylated proteins, plasma proteins and nanocapsules accumulate in tumors via the EPR effect (Attia et al., 2019). Physicochemical properties of the nano-DDS, including their surface charge, hydrophobicity/hydrophilicity, and biocompatibility, can affect the EPR effect. In addition, EPR-mediated accumulation in tumors is thought to be more pronounced in small animal tumor models than in human malignant tissues (Shi et al., 2020). A recent analysis revealed that a median of 0.7% of the injected dose of the nano-DDS reached the tumor by the EPR effect (Wilhelm et al., 2016). Hence, the extent and intensity of the EPR effect in human tumors is highly debatable (Shi et al., 2020).

### 2.2. Active targeting

**Figure 2.**
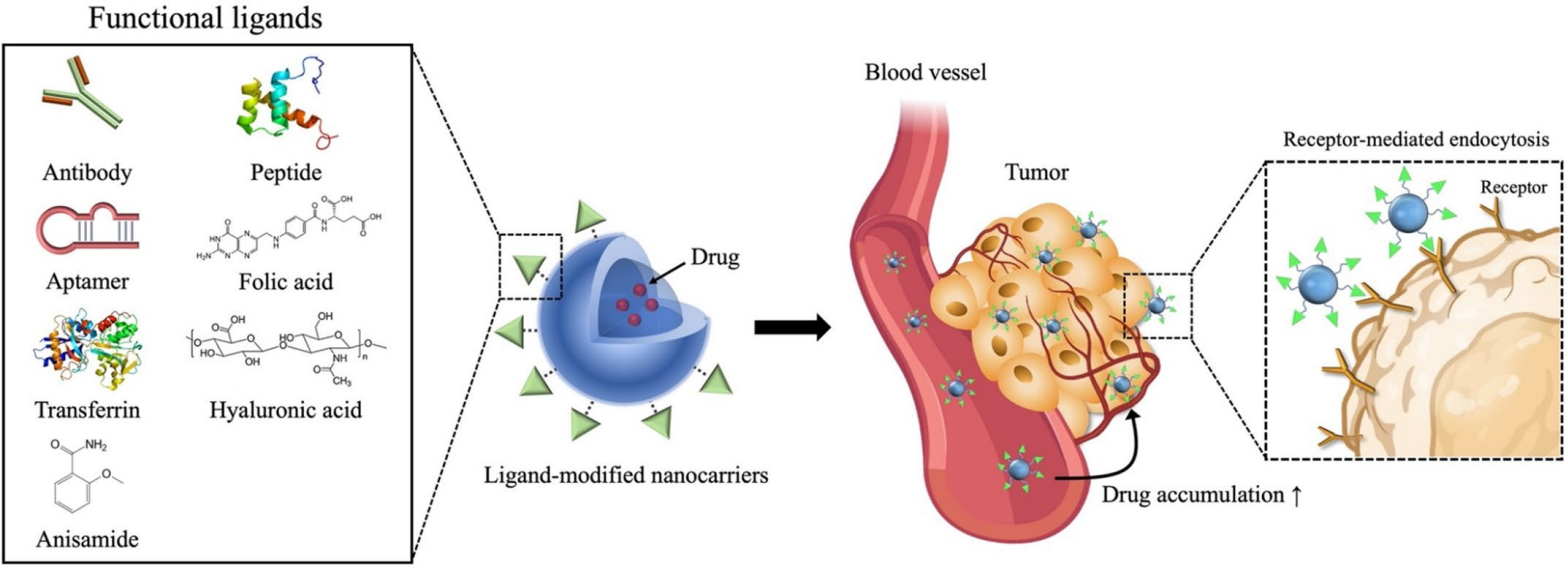
Strategy for active tumor-targeting via ligand-modified nanocarriers. Active targeting is achievable via surface modification of drug carriers with targeting ligands capable of interacting with antigens or receptors overexpressed (or present specifically) in tumors. Various functional ligands including folic acid, hyaluronic acid, transferrin, peptides, and antibodies, have been extensively explored to develop tumor-selective drug delivery systems.

The pathophysiological and micro-environmental differences between healthy organs and diseased organs play an important role in selective drug delivery to the site of action (Muhamad et al., 2018). In particular, active targeting relies on the differential expression of receptors and antigens between normal cells and pathologic cells (Riaz et al., 2018). Active targeting is achievable via surface modification of drug carriers with targeting ligands capable of interacting with antigens or receptors overexpressed (or present specifically) in tumors (Yoo et al., 2019). Therefore, tremendous efforts have been devoted to the identification of various cell surface receptors and the application of their specific ligands in designing a target-selective drug carrier (Figure 2). More details of these functional ligands and their applications for target-selective drug delivery are covered in the next section.

### 2.3. Functional ligands for tumor-targeted drug delivery

#### 2.3.1. Folic acid

Folic acid (FA) is a low molecular weight vitamin (B9) containing a pterine moiety and a glutamate entity linked by *p*-aminobenzoic acid. FA is essential for all eukaryotic cells for the biosynthesis of purines and pyrimidines and 1-carbon metabolism (Frigerio et al., 2019). As mammals are unable to synthesize folate by themselves, it has to be derived entirely from dietary sources (Ducker & Rabinowitz, 2017). Folate receptors (FRs) are cysteine-rich glycoproteins with molecular weights of 38–44 kDa, which transport folate into cells through the endocytosis process (Fernández et al., 2018). The human FR family comprises four isoforms –α, -β, -γ, and –δ as reported in the literature to date (Spiegelstein et al., 2000). FR-α, FR-β, and FR- δ are all attached to the cell membrane via glycosylphosphatidylinositol (GPI) anchors, whereas FR-γ lacks a GPI region and is a soluble secretory protein found only in hematopoietic cells. FR-δ has proven challenging to identify in human tissues, possibly due to a highly restricted spatial/temporal expression pattern, presence of a pseudogene, or predominance of an alternatively spliced variant (Tian et al., 2012).

FR-α is overexpressed in many epithelial cancers, including those of the ovary, breast, prostate, uterus, kidney, brain, and lung (Fernández et al., 2018; Scaranti et al., 2020). In addition, FR-α contributes to cell growth regulation and signaling functions in cancer malignancy (Cheung et al., 2016). The prevalence of FR-α overexpression among human cancers compared to healthy cells provides an opportunity to utilize FR-α as a target for tumor-selective drug delivery (Carron et al., 2018). FR-β shares nearly 70% of its homology with the FR-α isoform and both possess a comparable affinity for folate (Fernández et al., 2018). FR-β expression is elevated in activated myeloid cells (monocytes and macrophages) associated with inflammatory and autoimmune diseases (Fernández et al., 2018; Steinz et al., 2022). In addition, the FR-β expression level is amplified in most of the nonepithelial origin malignancies such as myelogenous leukemia and sarcoma (Roy et al., 2021). FR-α and FR-β are overexpressed in more than 40% and 25% of human cancers, respectively, and exhibit a high affinity to FA (Shen et al., 2015; Shen et al., 2018). In particular, FR-α isoform has a high affinity to FA (*Kd < 1 0 ^−9 ^M*) (Chandrupatla et al., 2019), which is crucial for tumor tissues to sustain their speedy and uncontrolled proliferation (Eales et al., 2016). The crystal structure of human FR-α in complex with FA revealed that the glutamate of FA sticks out of the binding pocket entrance, whereas the pteroate moiety is situated inside the binding pocket of FR-α (Chen et al., 2013). Accordingly, the derivatization of the α- and γ-carboxylic groups of the glutamate does not alter the binding affinity of FA to FR-α. Given that FA-conjugated molecules can bind effectively to FR-α and undergo receptor-mediated endocytosis, FA-conjugation should be an attractive approach to promote tumor-selective drug delivery. For example, EC145 (vintafolide) is a water- soluble folic acid derivative linked through a peptide spacer to the desacetylvinblastine monohydrazid (Lorusso et al., 2012). EC145 has entered phase 2 trials for several types of cancers (Scaranti et al., 2020). EC1456, a folate conjugate containing a highly cytotoxic tubulysin has been investigated in advanced solid tumors and ovarian cancer during clinical trials (Endocyte, 2015). In addition to direct conjugation to drug molecules, a wide range of drug carriers such as liposomes, micelles, polymers, dendrimers, and inorganic nanomaterials have been conjugated to FA and evaluated for their potential as a tumor-selective drug delivery system by using various anticancer drugs (e.g. doxorubicin, paclitaxel, docetaxel, 5-fluorouracil, erlotinib, curcumin, resveratrol, etc.). More details are well covered in previous reviews (Narmani et al., 2019; Ebrahimnejad et al., 2022).

#### 2.3.2. Hyaluronic acid

Hyaluronic acid (HA) is a natural polysaccharide that consists of repeating D-glucuronic acid and N-acetyl-D-glucosamine disaccharide units linked by (1-β-4) and (1-β-3) glucosidic bonds (Luo et al., 2019; Cho, 2020). The molecular weight (MW) of HA ranges from thousands to millions Daltons. Depending on its MW, HA exhibits distinct properties and plays different physiological roles. HA is widely distributed in the synovial and extracellular matrix and exhibits biocompatibility, biodegradability, high moisture retention, and tunable viscoelastic properties (Jiao et al., 2016). Due to its various biological effects including anti-inflammation, wound recovery, antiaging, tissue regeneration, and skin-repairing properties, HA has been extensively explored in the cosmetic and pharmaceutical industries (Narurkar et al., 2016; Bukhari et al., 2018).

HA is a ligand for the cluster-determinant 44 receptor (CD44) (Wickens et al., 2017; Cho, 2020). CD44 is a transmembrane glycoprotein with a molecular weight of 85–200 kDa, and it exists in various isoforms because of variable exon splicing and post-transcriptional modifications in gene expression (Azevedo et al., 2018; Chen et al., 2018). CD44 is vital in the release of cytokines, activation of lymphocytes, and regulation of hyaluronic metabolism in normal tissues (Senbanjo & Chellaiah, 2017).

Due to its tumorigenic functions and level of expression often associated with tumor-initiating cells/cancer stem cells, CD44 is considered an early indicator for cancer cell proliferation (Basakran, 2015; Rios De La Rosa et al., 2018). This establishes CD44 as a biomarker in cancer diagnosis, particularly for breast, colorectal, head and neck, pancreas, bowel and prostate cancers (Rios De La Rosa et al., 2018). CD44 has a high affinity for HA, and all isoforms of CD44 contain an HA-specific binding domain near the N terminus (Lintuluoto et al., 2021). Overexpression of CD44 in various cancer cells and its affinity for HA suggest a high potential for CD44 as a desirable target for enhancing tumor-selective drug delivery.

Conjugation of drug or drug-loaded nano-carriers with HA increases cancer cell uptake via CD44-mediated endocytosis (Huang et al., 2014a; Cho, 2020). Drug or drug-loaded nano-carriers can be linked with HA through amidation, esterification, oxidation, etherification, cross-linking, grafting, and Ugi condensation (Jiao et al., 2016). Apart from the targeting capacity, HA prolongs the blood circulation of nanoparticles. HA can also reduce the immunogenicity of nano-carriers and serves as an alternative for polyethylene glycol (PEG) employed for the same purpose (Almalik et al., 2017). Owing to many advantages, HA has been extensively employed in nano-drug delivery systems. For example, the surface coating of curcumin-loaded zein nanoparticles with HA improved the distribution of curcumin in CD44-overexpressed CT26 tumor cells and enhanced the anticancer effect (Seok et al., 2018). Lv et al. (2018) prepared HA-PTX/MATT-LTSL HNPs by modifying marimastat (MATT)-loaded thermal-sensitive liposomes with paclitaxel (PTX)-HA prodrug. These hybrid nanoparticles significantly hampered 4T1 tumor growth, metastasis and tumor cell angiogenesis (Lv et al., 2018). Also, the phase 1 clinical trial of HA-irinotecan demonstrated that this conjugate achieved safety and tolerance without compromising the anticancer activity of irinotecan (Gibbs et al., 2011).

#### 2.3.3. Transferrin

Transferrin (Tf) is a monomeric glycoprotein with a molecular mass of 78 kDa that consists of 679 amino acids. The Tf structure is composed of two structurally similar globular domains, known as the N- and C- terminal domains, and connected by a short linear peptide linker. The N- and the C-domains are composed of α-helices and β-sheets and can be further divided into the subdomains; N1, N2, C1, and C2. Each domain contains an iron-binding site, which has a very high binding affinity (KD = 10 − 22 M) for Fe^3+^ ion (Luck & Mason, 2013). Tf undergoes a conformational change when it binds to iron, which, in turn, is important for its selective recognition by Tf receptors (Sharma et al., 2013). There are two transferrin receptors known as TfR1 and TfR2 and both are type II transmembrane glycoprotein (Leitner & Connor, 2012). The human TfR1 is involved in iron uptake and cell growth regulation and consists of two identical subunits of 90 kDa each linked by two disulfide bonds (Sharma et al., 2013). TfR1 is able to internalize two Tf via TfR-mediated endocytosis (Anderson & Vulpe, 2009). While TfR2 shares 66% homology with TfR1, TfR1 has a 25-fold higher affinity to Tf than TfR2 (Kawabata et al., 2000). TfR1 is highly expressed in some immature erythroid cells with a large requirement of iron for hemoglobin synthesis, vascular endothelium of brain capillaries, and actively proliferating cancer cells (Johnsen et al., 2019; Koneru et al., 2021). In particular, high affinity to Tf and overexpression in cancer cells make TfR1 an attractive target for improving the efficiency of chemotherapy.

Jose et al. (2019) demonstrated that the anticancer activity of the Tf-conjugated PLGA nanoparticles (NPs) loaded with docetaxel was very promising because of their ability to arrest cancer activity at the G2/M phase of mitosis (Jose et al., 2019). Cui et al. (2017) also prepared Tf-conjugated magnetic PLGA NPs containing PTX (PTX-MNP-PLGA NPs). They demonstrated that Tf-conjugated PTX-MNP-PLGA NPs had greater antiproliferation and higher cellular uptake in U-87 cells, compared to unmodified NPs or free PTX (Cui et al., 2017). Some other previous studies also suggest that Tf-conjugated NPs, including polymeric NPs, or liposomes, have a high potential for targeting gliomas (Lv et al., 2013; Sun et al., 2017).

#### 2.3.4. Antibodies

Antibodies and their fragments have been broadly exploited for targeted drug delivery due to their high specificity and affinity for cognate antigens. The structure of antibodies allows for specific interactions between therapeutic targets and the immune system. By binding to a target antigen, antibodies can neutralize it, preventing undesirable cellular processes (Chiu et al., 2019). Additionally, antibodies can interact directly with host immune cells to initiate phagocytosis, antibody-dependent cellular cytotoxicity or complement-dependent cytotoxicity, triggering cell death (Hoppenz et al., 2020). So far, five antibody–drug conjugates have received regulatory approval from the US Food and Drug Administration (FDA) and the European Medicines Agency (EMA) (Hoppenz et al., 2020). Among them, trastuzumab emtansine and brentuximab vedotin are on the market (Srinivasarao et al., 2015).

Targeted drug delivery can be achieved via the modification of drug carriers with antibodies or antibody fragments. Manjappa et al. (2014) conjugated Fab’ fragments of neuropilin-1 antibody to PEGylated liposomes containing docetaxel via thioether linkage, producing stable and efficient anti-neuropilin-1 immunoliposomes. These antibody-functionalized immunoliposomes demonstrated higher *in vivo* tumor suppression in the mouse model than non-functionalized liposomes and commercial products, suggesting the neuropilin-1 antibody fragment as a potent ligand for targeting the neuropilin-1 receptor in cancer cells (Manjappa et al., 2014). While the unique *in vivo* properties and high target specificities of antibody-based targeting ligands have gained great attention, the lack of reliable chemistry to attach antibodies to the drug carriers and the potential immunogenicity of antibodies have limited their clinical applications (Jiang et al., 2019). Fc fragments can direct the fast clearance of drug carriers by activating mononuclear phagocytic systems. In addition, the large size, poor permeability, high cost, and reduced product homogeneity due to nonselective payload conjugation are other limiting factors for the application of antibodies in drug delivery (Jiang et al., 2019).

#### 2.3.5. Aptamers

Aptamers are single-stranded DNA or RNA oligonucleotides that can bind to specific target substances, including drugs, proteins, and receptors. They possess some unique properties, including small size (15 kDa), biodegradability, low immunogenicity, a simple and rapid synthetic process, low cost, high specificity, and ease of labeling (Jiang et al., 2019). Due to these favorable characteristics, it has gained attention as a good ligand for cancer cell targeting. They have a specific binding ability to cancer-related biomarkers and cancer cells by folding into well-defined three-dimensional structures (Huang et al., 2021) and thus provide a promising way to deliver imaging agents and drugs to tumors. Furthermore, the fabrication of aptamer is executed out of the biological systems, reducing the risk of bacterial or viral contaminations (Huang et al., 2021; Shigdar et al., 2021). To date, isolation of various aptamers has been accomplished to specifically target substances in cancer cells, including mucin 1 (MUC1), epithelial cell adhesion molecule (EpCAM), platelet-derived growth factor (PDGF), vascular endothelial growth factor (VEGF), nuclear factor-kB (NF-kB), programmed death-ligand 1(PDL1), and prostate-specific membrane antigen (PSMA) (Hashemi et al., 2020). Aptamers are similar to antibodies in terms of their high sensitivity and specificity as targeting agents. However, aptamers have higher tumor penetration, retention, and homogenous distribution, compared to antibodies (Cerchia, 2018). The smaller size of aptamers leads to enhanced tumor penetration and also has them attached to the surfaces of nanoparticles with higher density without steric hindrance (Moosavian & Sahebkar, 2019). Accordingly, the attachment process of aptamers to the surface of nanoparticles is more amenable and reproducible than antibodies. Owing to these features, aptamers are considered promising ligands for active tumor targeting (Moosavian & Sahebkar, 2019).

Taghavi et al. (2017) fabricated chitosan-modified PLGA nanoparticles tagged with the 5TR1 aptamer, demonstrating the enhanced antitumor activity of aptamer-tagged nanoparticles. Recently, co-delivery of epirubicin and antimir-21 via MUC1 aptamer-modified PLGA nanocomplex showed enhanced antitumor activity in tumor-bearing mice when compared with epirubicin alone and other treatments (Bahreyni et al., 2019). Upon binding to the extracellular domain of the proper targets, aptamers undergo receptor-mediated endocytosis, driving the internalization of therapeutic agents. In a recent study, Lv et al. (2019) used the single-particle tracking (SPT) technique to monitor in real time the specific endocytic pathways and intracellular transport of sgc8, a DNA aptamer that targets the protein tyrosine kinase 7 (PTK7). By conjugating the sgc8 aptamer to the 5-fluorouracil (sgc8-5FU), they demonstrated that, upon binding to PTK7, the aptamer, either alone or in the context of the conjugate, is internalized mainly via caveolin-mediated endocytosis, although partially via clathrin-mediated endocytosis.

Although aptamers exhibit excellent performance *in vitro*, there are still many bottlenecks in clinical application. For example, aptamers are easily affected by external substances such as nonspecific serum binding proteins, resulting in the reduced binding efficiency to target materials. Degradation during blood circulation also limits the application of aptamers (Lakhin et al., 2013). The inherent physicochemical properties need to be improved, and a better understanding of their pharmacokinetics, pharmacodynamics, and potential toxicity is necessary to ensure the clinical success of aptamers (He et al., 2020).

#### 2.3.6. Anisamide

Anisamide is a low-molecular-weight benzamide derivative and can be utilized as a tumor-directing moiety in functionalized drug delivery systems via its alleged interaction with sigma receptors (Dasargyri et al., 2017). Sigma receptors (σ1 and σ2) are transmembrane proteins and play a role in regulating ion channels (Van Waarde et al., 2015). Sigma receptors are overexpressed in human tumors, including breast cancer and prostate cancer (Van Waarde et al., 2015). Owing to the high affinity of anisamide for sigma-receptors, surface modification of drug carriers with anisamide has drawn great attention for active tumor targeting. Luan et al. (2019) fabricated anisamide-functionalized gold nanoparticles to target prostate cancers, which were effectively complexed with siRNA *via* electrostatic interaction. The obtained anisamide-targeted gold nanoparticles complexed with siRNA selectively bound to human prostate cancer PC-3 cells, inducing efficient endosomal escape of siRNA (Luan et al., 2019). Furthermore, the anisamide-targeted gold nanoparticles prolonged the systemic exposure of siRNA, leading to significant tumor growth suppression in a xenograft mouse model without increasing toxicity (Luan et al., 2019). Recently, Yao et al. (2021) also reported that the anisamide-modified dual-responsive liposomes with MRI capacity can provide a powerful tool for cancer targeting therapy.

However, given that the antitumor efficacy of the anisamide-decorated drug delivery systems varies considerably in previous studies and the interaction of anisamide with the sigma receptors is not clearly defined yet, further research is necessary to elucidate more clearly the utility of anisamide as a ligand for tumor targeting (Dasargyri et al., 2017).

#### 2.3.7. Peptides

Peptide ligands have many advantages including the accessibility of high-throughput screening, ease of synthesis and manipulation, high specificity and affinity to a diverse range of targets, and less immunogenicity (Jiang et al., 2019). These peptides are also called cell targeting peptides or more commonly tumor targeting/tumor homing peptides (Kondo et al., 2021). Peptide ligands used for targeted drug delivery are mainly identified via bio-inspired techniques (biomimetic peptides) or large-scale screening of peptide libraries, including phage display peptide libraries and chemical peptide libraries (Huang et al., 2014b; Jiang et al., 2019). This class of peptide ligands has a variety of origins, structures, targets, and biomedical applications, providing vast resources for achieving targeted drug delivery (Huang et al., 2021; Liu et al., 2021). Numerous receptors that are involved in the development and progress of cancers have been found to interact with naturally occurring proteins or peptides. Many naturally occurring proteins and peptides are potent ligands for their receptors; however, their direct applications for targeted delivery are restricted by various issues, such as low biocompatibility, poor specificity, high toxicity, and large size (Jiang et al., 2019). However, structure-based peptide optimization can be conducted to identify biomimetic peptide ligands that can overcome the shortcomings of natural peptides and provide advantages such as high stability, enhanced specificity and affinity, and lack of toxicity (Jiang et al., 2019). For instance, octreotide (SMS 201-995), synthetic analogue of somatostatin, has been applied for the targeted delivery of radiotherapeutic agents, chemotherapeutic agents, liposomes, and micelles (Jiang et al., 2019). In recent years, many efforts have been made to develop biomimetic peptides from the toxin that are applicable to targeted drug delivery. For example, KC2S peptide is a biomimetic peptide that can specifically bind to nicotinic acetylcholine receptors (nAChRs) with high affinity by mimicking the loop 2 segment of *Ophiophagus hannah* toxin b (KC2S). This peptide exhibited potent brain targeting ability when modified on the surface of PEG-PLA micelles. However, the disulfide bond in KC2S that is necessary for receptor binding is prone to reduction in blood (Zhan et al., 2010). To overcome this issue, CDX (a shorter biomimetic peptide without this disulfide bond) was developed using a computer-aided peptide design (Zhan et al., 2011). CDX peptide is derived from candoxin, a three-finger snake neurotoxin isolated from *Bungarus candidus*. The surface modification of nanocarriers with CDX dramatically improved the anti-glioma effects of paclitaxel-loaded micelles in nude mice (Zhan et al., 2011).

In contrast to structure-based biomimetic design, phage display allows for the development of peptide ligands without knowledge of their binding properties; this has resulted in the rapid discovery of new peptide ligands (Aloisio et al., 2021). Several peptide motifs that target cancer cells have been discovered using phage display in the past decades, as exemplified by NGR (asparagine-glycine-arginine) and RGD (arginine-glycine-aspartic acid) (Saw & Song, 2019). RGD targets integrins that are heterodimeric transmembrane glycoproteins overexpressed in the endothelial cells of tumor vasculature (Nieberler et al., 2017). Integrins are composed of α and β subunits, which determine the binding affinity of integrin-associated ligands. The RGD peptide is capable of recognizing and binding specifically to αvβ3 and αvβ5 integrins, facilitating its tumor vasculature accumulation (Nieberler et al., 2017). Recently, Lv et al. (2020) developed self-assembled nanoparticles of BAK (pro-apoptotic protein) targeting integrin α_v_β_3_ overexpressed tumor cells. In their studies, recombinant proteins with BH3 BAK as an active domain and RGD peptides as targeting ligands were self-assembled into protein nanoparticles. The results showed that RGD-decorated nanoparticles inhibited the proliferation of cancer cells, with a lower IC_50_ than the nanoparticles without RGD peptides, suggesting a potential of RGD peptides as a ligand for tumor targeting.

Numerous NGR-containing peptide sequences have been recognized and optimized through phage-display libraries, which can bind to CD13/aminopeptidase N expressed in tumor vasculature (Enyedi et al., 2017). The cyclic forms of NGR peptides show greater selectivity than linear forms (Enyedi et al., 2017; Zhu et al., 2020). A cyclic NGR peptide (CNGRC) has been applied for targeted delivery of various anticancer drugs (e.g. doxorubicin, docetaxel, carboplatin, 5-fluorouracil prodrug, and 5-fluoro-2′-deoxyuridine prodrug) and siRNA (Enyedi et al., 2017; Zhu et al., 2020). Since NGR-peptides are able to bind to both CD13 and RGD binding integrin, they are considered ideal candidates for selective and dual-acting ligands (Enyedi et al., 2017). Recently, Seidi et al. (2018) developed a novel bi-functional protein consisting of truncated coagulase (tCoa) and an NGR (GNGRAHA) motif for targeting both CD13 and αvβ3 integrin-positive tumor neovasculature. The systemic administration of tCoa-NGR significantly reduced tumor growth in mouse models (Seidi et al., 2018).

Peptide **1** (or GE11) is a dodecapeptide that binds specifically to the epidermal growth factor receptor (EGFR or ErbB1) overexpressed in a number of tumors of epithelial origin including breast cancer, and is being used as a cancer cell-targeting peptide (Genta et al., 2017). Peptide **1** was identified by screening a phage display peptide library against purified human EGFR protein (Hossein-Nejad-Ariani et al., 2019). Hossein-Nejad-Ariani et al. (2019) designed a synthetic peptide library of 29 analogues of peptide **1** (GE11) with the aim of targeting EGFR, specifically in triple-negative breast cancer (TNBC) cells. By using a whole cell-peptide binding assay and flow cytometry, they identified several new analogues of peptide **1** with higher binding and uptake to TNBC cells. These new analogues of peptide **1** targeting EGFR can be used for the targeted delivery of chemotherapeutics like doxorubicin in the treatment of TNBC (Hossein-Nejad-Ariani et al., 2019).

Overall, it is expected that drug carriers decorated with tumor vasculature-homing peptides should facilitate tumor-targeted drug delivery, improving the effectiveness of chemotherapy with less side effects.

## 3. Approaches for enhancing intracellular delivery of anticancer drugs

**Figure 3.**
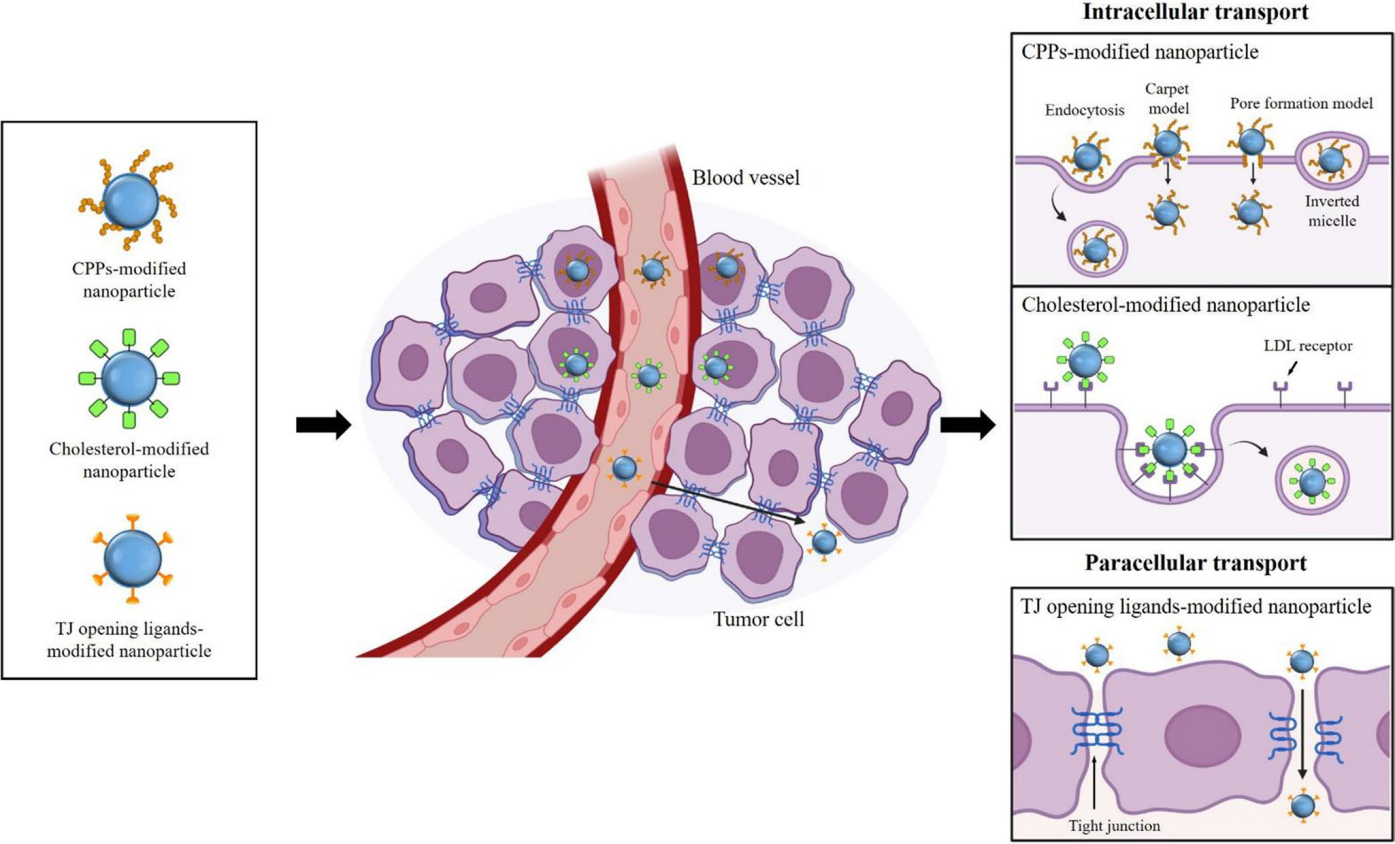
Strategy for enhancing intracellular uptake of anticancer drugs. Ligand-grafted nanoparticles can enhance the transcellular and paracellular transport of anticancer drugs. Surface modification of nanoparticles with cell-penetration peptides (CPPs) improves the intracellular drug uptake via multiple pathways including the energy-dependent endocytosis and direct translocation pathways. Cholesterol can be also utilized as a targeting ligand to increase the intracellular drug uptake via low-density lipoprotein (LDL) receptor-mediated endocytosis. In addition, incorporation of tight junction opening ligands to nanoparticles is applicable to enhance the paracellular drug transport.

Anticancer drugs should undergo intracellular uptake in cancer cells prior to exerting therapeutic efficacy and thus the limited penetration of drugs, nanoparticles, or poor accessibility to cancer cells remains a big challenge in chemotherapy. Various approaches have been attempted to improve the intracellular delivery of drugs and drug-loaded carriers. Among them, as illustrated in Figure 3, the utilization of functional ligands to improve the cellular uptake of anticancer drugs is briefly introduced in the next section.

### 3.1. Cell-penetrating peptides

Cell-penetrating peptides (CPPs) are short peptides with 5–30 amino acids (Langel, 2011). Due to their ability to penetrate the cell membranes and low cytotoxicity, CPPs have gained great attention in recent decades (Dissanayake et al., 2017; Silva et al., 2019). Since Frankel and Pabo (1988) discovered the human immunodeficiency virus (HIV) trans-activator of transcription (TAT) protein in 1988, many new CPPs have been discovered for the effective delivery of diverse therapeutics, including nucleic acids, proteins, and chemotherapeutic agents. As the initial CPPs were natural peptides derived from protein fragments, such peptides were also described as the protein transduction domain (PTD). Recently, many CPPs with differences in charge, polarity, and/or structure have been discovered and/or designed. In particular, combinatorial peptide chemistry and orthogonal high-throughput screening expedite the identification of spontaneous membrane-translocating peptides (Marks et al., 2011). A CPP database (http://crdd.osdd.net/raghava/cppsite/) established in 2012 contains about 1855 kinds of CPP sequences and will be expanded continuously. CPPs have high diversity in physicochemical and biological properties (Xie et al., 2020). Some of the CPPs are exampled in Table 1.

**Table 1.** Selected examples of CPPs based on physicochemical properties (Derakhshankhah & Jafari, 2018; Xie et al., 2020).

Type	CPPs	Amino acid sequences
Cationic	R8	RRRRRRRR
	TAT	GRKKRRQRRRPQ
	R9-TAT	GRRRRRRRRRPPQ
	Penetratin	RQIKIWFQNRRMKWKK
	DPV3	RKKRRRESRKKRRRES
	DPV6	GRPRESGKKRKRKRLKP
Anionic	Azurin-p28	LSTAADMQGVVTDGMASGLDKDYLKPDD
Amphipathic	ARF (19-31)	RVRVFVVHIPRLT
	MAP	KLALKLALKALKAALKLA
	pVEC	LLIILRRRIRKQAHAHSK
	MPG	GALFLGFLGAAGSTMGAWSQPKKKRKV
	Transportan	GWTLNSAGYLLGKINLKALAALAKKIL
	Pep-1	KETWWETWWTEWSQPKKKRKV
Hydrophobic	Bip4	VSALK
	C105Y	CSIPPEVKFNPFVYLI
	gH625	HGLASTLTRWAHYNALIRAF
	Melittin	GIGAVLKVLTTGLPALISWIKRKRQQ

Since CPPs are able to carry various cargoes without cellular injury, they have been used to promote the intracellular delivery of drug molecules, nucleotides, proteins, and peptides (Chang et al., 2014). In addition, nanocarriers modified with CPP can achieve enhanced cellular uptake in tumors. For example, PEGylated docetaxel nanocrystals modified with TAT enhanced cellular drug uptake, leading to stronger cell growth inhibition in cervical cancer-related TC-1 cells when compared to PEGylated nanocrystals without TAT (Lv et al., 2021).

The exact mechanism of CPP-mediated transport across biological membranes is still unknown. The efficiency and transport mechanism of CPP can be influenced by various factors, including CPP sequence, amphipathicity, charge, cell type, and cargo (Kauffman et al., 2015). Based on the energy requirement for the process of internalization, the cellular uptake pathways of CPPs have been generally divided into two categories: the energy-dependent endocytosis and the energy-independent non-endocytic uptake mechanism (Xie et al., 2020). For the large molecular weight CPPs or CPP/cargoes, the endocytosis is the principal cellular uptake mechanism (Yang et al., 2019).

The application of CPPs as carriers for the delivery of biopharmaceuticals across the intestinal epithelium is pursued via covalent conjugation of CPPs to drugs or drug carriers or via simple co-administration as a physical mixture that exploits the weak interaction between CPPs and cargos (Kristensen & Nielsen, 2016). Given that CPPs can be internalized by almost all types of cells, there might be cellular toxicity caused by off-target cellular absorption of the therapeutics by normal tissues. As a result, dual functionalized drug carriers attached with CPPs and cancer cell targeting ligands should be promising to promote cancer cell-specific drug delivery and subsequent internalization of anticancer drugs at the site of action. This dual functionality may reduce the undesired side effects by compensating for the nonselectivity of CPPs. For example, Salehi Khesht et al. (2021) developed chitosan lactate nanoparticles functionalized with HIV-1-derived TAT peptide and hyaluronate to co-deliver doxorubicin and CD73 siRNA to cancer cells. These dual functionalized nanoparticles significantly suppressed the angiogenesis, invasion, proliferation, and migration of cancer cells (Salehi Khesht et al., 2021). Recently, a ‘smart’ intracellular drug delivery system based on CPP was designed and named ‘ATTEMPTS’ (antibody-targeted triggered electrically modified prodrug type strategy) (Xie et al., 2020). This system has two components: one is antibodies-conjugated heparin for targeting, and the other is the CPP-modified drug component. These two components form a compact complex by electrostatic adsorption between cationic CPP and the anionic heparin (Xie et al., 2020). The electrical charge of CPPs is neutralized by heparin, increasing the plasma stability of CPP/cargo complex against endogenous proteases (Xie et al., 2020). After intravenous injection, the antibody will carry the whole complexes to the target site, and then the clinical heparin antidote protamine sulfate is systemically injected to separate the CPP-modified drug from the complex since protamine has a stronger heparin-binding affinity than CPPs. Subsequently, the released CPP-modified drug could be internalized into the tumor cells (Xie et al., 2020).

### 3.2. Cholesterol

Cholesterol is a neutral lipid that plays a vital role in the maintenance of the integrity of biological membranes and serves as a precursor in the synthesis of many endocrine mediators (Tong, 2011). Cholesterol is biocompatible, biodegradable, highly permeable, readily available, and inexpensive (Ruwizhi & Aderibigbe, 2020). Its functional groups are also easily derivatized. The incorporation of cholesterol to drugs or drug carriers can enhance their biological activities, stability, or cellular uptake (Ruwizhi & Aderibigbe, 2020; Tedesco et al., 2021). Accordingly, chemical modification of drug carriers with cholesterol has been attempted in various delivery systems, including nanoparticles, micelles, niosomes, and liposomal formulations (Ruwizhi & Aderibigbe, 2020). Cholesterol-modified carriers have the ability to incorporate both lipophilic and hydrophilic drugs with high drug loading efficiency and could enhance the intracellular uptake (Muddineti et al., 2018; Nakhaei et al., 2021). Muddineti et al. (2018) developed cholesterol-modified low molecular-weight chitosan for co-delivery of siRNA and curcumin to cancer cells. The cholesterol-modified chitosan micelles exhibited optimum physicochemical characteristics, dual drug loading capability, and enhanced intracellular drug uptake (Muddineti et al., 2018).

In addition to enhancing cellular uptake, cholesterol can be utilized as a targeting moiety in cancer chemotherapy. For example, the high requirement for low-density lipoprotein (LDL) by malignant cells and the overexpression of LDL receptors in cancer cells provide an opportunity to utilize LDL as a ligand for tumor-selective drug delivery (Radwan & Alanazi, 2014). Many types of tumor cells display a higher level of receptor mediated-LDL uptake compared to corresponding normal tissues, which is probably due to high cholesterol demand for cell growth and/or a mechanism directly linked to cell transformation (Radwan & Alanazi, 2014; Markovic et al., 2020). Therefore, LDL has been proposed as a potential ligand for the targeted delivery of chemotherapeutic agents (Radwan & Alanazi, 2014). Cholesterol has also been conjugated with imaging molecules to develop an efficient theranostic system for cancer treatment. Tedesco et al. (2021) developed a cholesterol-rich nanoemulsion called LDE containing aluminum phthalocyanine chloride, a highly fluorescent second-generation photosensitizer, to treat cancer cells with high expression of LDL receptors. Anticancer drugs can also be directly conjugated with cholesterol molecules, to improve their pharmacokinetic behavior, cellular uptake, target specificity, and safety (Radwan & Alanazi, 2014; Chen et al., 2015; Ruwizhi & Aderibigbe, 2020).

Collectively, due to the capability for tumor targeting and also enhancing cellular uptake, conjugation of cholesterol to drugs or drug carriers provides a promising approach to enhance the therapeutic efficacy with low toxicity in cancer chemotherapy.

### 3.3. Ligands for tight junction opening

The therapeutic efficacy of anticancer drugs in solid tumors depends on the drug’s penetration into the tumor mass, affinity to the target, and retention at the site of action (Moradi Kashkooli et al., 2021). Epithelial cancers are characterized by tight junctions (TJs) that create obstacles to naturally occurring immune cells, antibodies, and drug therapies (Pitner et al., 2019). Studies have revealed a positive correlation between the upregulation of TJ proteins in solid tumors and their resistance to drug therapy (Martin, 2014; Pitner et al., 2019). The primary function of TJs is to mediate intercellular adhesion and polarity. Intercellular TJs act as a formidable barrier against paracellular drug absorption. TJs comprise transmembrane proteins, including occludin, claudins, junctional adhesion molecules, and tricellulin as well as intracellular scaffold proteins like zonula occludens (ZO) and cingulin (Salvador et al., 2016). TJ proteins have enormous potential to inhibit tumorigenesis via the promotion of stable cell–cell adhesion or to support tumorigenesis via adhesion-independent signal transduction events that govern migration, proliferation, and apoptosis (Leech et al., 2015). Furthermore, several cancers have altered the expression of TJ proteins, making them attractive diagnostic and prognostic markers (Martin, 2014; Leech et al., 2015).

Since modulation of TJ proteins can allow the enhanced bioavailability and organ deposition of drugs (Brunner et al., 2021), there have been great efforts to discover effective modulators for TJ proteins. In particular, target-specific tight junction openers should be promising candidates as antitumor adjuvants (Brunner et al., 2021). The biopolymer chitosan, derived from the ubiquitous chitin, and its derivatives, have been the most studied nonspecific permeation enhancers. Different mechanisms have been suggested to explain the ability of this polymer to increase membrane permeability. The disruption of the lipid organization of the cell membrane and/or the interaction with TJ components were among the first hypotheses to explain the TJ modulation activity of chitosan (Lee et al., 2016; Brunner et al., 2021).

Recently, 3-aminopropyl functionalized magnesium phyllosilicate (aminoclay) was reported as a promising drug carrier having a reversible TJ opening effect (Lee et al., 2020; Song et al., 2021). Aminoclay is a synthetic organic–inorganic layered material that is delaminated to form water-soluble cationic nanosheets. It can interact with negatively charged drug molecules via electrostatic interaction to produce a drug–clay complex, thereby improving the stability, water solubility, and bioavailability of drug candidates. Furthermore, previous studies have demonstrated that aminoclay-based nanoparticles could modulate the tight junctions of Caco-2 cells, thus causing an increase in the para-cellular permeability (Lee et al., 2020; Song et al., 2021). Importantly, the effect of aminoclay-based nanoparticles on the tight junction opening appeared to be reversible.

The junction opener (JO) is a recombinant protein derived from adenovirus serotype 3 that can transiently open intercellular junctions in epithelial tumors by cleaving the junction protein desmoglein-2 (Wang et al., 2018). It contains the nominal structural domains essential for opening intercellular junctions. Co-administration of JO-1 was previously shown to facilitate the intratumoral penetration and therapeutic efficacy of monoclonal antibodies (mAbs) such as the anti-Her2/neu mAb trastuzumab (Herceptin) and the EGFR inhibitor cetuximab (Erbitux) (Beyer et al., 2011; Wang et al., 2018). Furthermore, JO-1 was tested in combination with several chemotherapeutic drugs, including paclitaxel (Taxol), irinotecan (Camptosar), nanoparticle albumin-bound paclitaxel (Abraxane), and liposomal doxorubicin (Doxil) (Beyer et al., 2011; Beyer et al., 2012; Wang et al., 2018). JO-1 co-therapy enhanced the efficacy of these drugs and overcame drug resistance in several tumor models while reducing the drug doses necessary for therapeutic effect (Beyer et al., 2012). JO-1 and variants of this protein (such as the affinity-enhanced version, JO-4) are, therefore, interesting for clinical application (Wang et al., 2018). Recently, Pitner et al. (2019) developed a new class of tumor-targeting protein (from JO-4), junction opener conjugated to *x* (JOC-x) that can accumulate both around and within tumors and remodel the tumor microenvironment.

RMP-7 is a synthetic bradykinin (a short-lived peptide hormone) analog that has a high binding affinity for the blood–brain barrier (BBB) and the capability to increase tight junction permeability (Emerich et al., 1999; Gao & Gao, 2018). In a rat glioma model, the intravascular administration of RMP-7 increased the delivery of carboplatin by more than two-folds and improved the survival (Emerich et al., 1999). As a result, RMP-7 incorporated-nanoparticles can provide a benefit for enhancing the drug penetration across the BBB and the blood–tumor barrier (BTB) (Gao & Gao, 2018). Kuo and Tsao (2017) have indicated the minor tight junction opening via the surface modification of liposomes with RMP-7 (Kuo & Tsao, 2017). Similarly, C-CPE, the C-terminal fragment of *Clostridium perfringens* enterotoxin, binds to the TJ protein claudin and promotes paracellular drug delivery (Black et al., 2015; Saitoh et al., 2015; Kojima et al., 2016; Becker et al., 2018).

Overall, the conjugation of TJ opening ligands might be a promising strategy for enhancing the delivery of various anticancer drugs or drug carriers into the tumor mass, subsequently enhancing the effectiveness of chemotherapy. More detailed information on various strategies for TJ opening is reviewed elsewhere (González-Mariscal et al., 2016).

## 4. Summary and future perspectives

Cancer chemotherapy with cytotoxic small molecules or biologics is often limited by the off-target toxicity and poor penetration into the tumor mass. Consequently, optimization of both selectivity to cancer cells and accessibility to the intracellular target is critical to improving the therapeutic efficacy and quality of life of patients undergoing chemotherapy. Various functional ligands have been identified and characterized to achieve the selective delivery of anticancer drugs to their targets. Although direct conjugation of these functional ligands to drug molecules, including pharmacophores, is available, it may alter the biological activity of drugs. Therefore, the surface modification of drug carriers with these functional ligands has been more actively pursued by a wide range of anticancer drugs. The utilization of functional ligands appears to be successful, for a certain extent, but a single ligand is not able to overcome both functional and physical barriers so as to safely and selectively deliver drugs to the intracellular targets. Specific targeting ligands alone do not guarantee the efficient intracellular uptake of anticancer drugs in tumors. Hence, along with the targeted approach, efficient intracellular trafficking is also essential to enhance the therapeutic effectiveness. Therefore, the incorporation of multiple ligands with different roles to prepare the multifunctional drug carriers should be more advantageous, compared to the adoption of a single ligand. The clinical success of these multifunctional drug carriers depends on many factors, including physicochemical properties (e.g. size, charge, rigidity, etc.), pharmacokinetic profiles, biological stability, drug release properties, cytotoxicity, and endosomal escape. The inherent heterogeneity of tumors (e.g. composition, structure, function, heterogeneity in the expression of targets, etc.) also affects *in vivo* performance and effectiveness of ligand-modified delivery systems. Furthermore, reproducibility of preparation methods, ease of large-scale up, and cost-effectiveness should be crucial for industrial production. Recently, various computational tools and predictive models are utilized for a better understanding of ligand–target interaction and also for a better designing of ligand-modified drug delivery systems. Given that the ligand-based delivery systems suitable for industrial production have enormous potential to improve the clinical application of various anticancer drugs, continuous efforts should be directed to identify additional new ligands and their derivatives, opening a new platform for the sophisticated and precise delivery of anticancer drugs to intracellular targets.
